# *OsPM19L* Coordinates Phytohormone Signaling to Regulate Axillary Bud Outgrowth and Regeneration in Ratoon Rice

**DOI:** 10.3390/plants14243843

**Published:** 2025-12-17

**Authors:** Ruoxi Li, Binbin Chi, Wei Su, Jing Chen, Tianle Li, Hao Ma, Langtao Xiao

**Affiliations:** 1 State Key Laboratory of Crop Genetics and Germplasm Enhancement, Nanjing Agricultural University, Nanjing 210095, China; liruoxi@stu.njau.edu.cn (R.L.); suwei@stu.njau.edu.cn (W.S.); cjchenjing@stu.njau.edu.cn (J.C.); litianle@stu.njau.edu.cn (T.L.); 2 Hunan Provincial Key Laboratory of Phytohormones and Growth Development, Hunan Agricultural University, Changsha 410128, China; cbb1438@gmail.com

**Keywords:** ratoon rice, axillary bud regeneration, ABA signaling, hormonal regulation

## Abstract

Ratoon rice cultivation is an efficient production system that achieves a second harvest from the stubble of the main crop, but its yield potential is largely constrained by variation in axillary bud regeneration capacity. Here, we identify *OsPM19L*, a plasma membrane–localized AWPM-19 domain protein, as a key regulator of rice ratooning ability. Transcriptome analysis revealed higher *OsPM19L* expression in strong-regeneration cultivars, followed by a sharp decline after harvest. Promoter assays and hormonal treatments demonstrated that *OsPM19L* is strongly induced by ABA and functions as a positive regulator in ABA signaling. Under field conditions, *ospm19l* mutants exhibited increased tiller number but reduced ratooning index, whereas *OsPM19L-OE* plants showed the opposite pattern, indicating stage-specific regulation of tillering and regeneration. Hormone profiling and gene expression analyses showed that *OsPM19L* is associated with altered levels of multiple phytohormones in regenerating axillary buds, showing higher CK and GA levels and lower IAA and ABA levels in *OsPM19L-OE* compared with the wild type. Consequently, *OsPM19L* appears to facilitate dormancy release and enhance early axillary bud growth during the ratoon season. These findings indicate *OsPM19L* may act as a central regulator linking ABA signaling with hormonal cross-talk, providing new insights into the molecular control of regeneration and potential targets for improving ratoon rice productivity.

## 1. Introduction

Rice is one of the most essential staple foods worldwide [1]. In recent decades, rapid urbanization and socioeconomic transformation have driven substantial rural-to-urban migration, resulting in pronounced agricultural labor shortages, particularly across southern rice-growing regions. This demographic shift has imposed growing pressure on conventional rice production systems, underscoring the urgent need for cultivation models that demand less manual input [2]. In this context, ratoon rice cultivation has emerged as a labor-efficient and resource-conserving alternative, enabling two harvests from a single planting while maintaining relatively low production costs [3]. Yet, despite its agronomic and economic advantages, the large-scale adoption of ratoon rice remains constrained by the lack of specialized varieties, leading to low and unstable second-crop yields [4]. This instability primarily arises from the limited ecological adaptability, low seed-setting rate, and weak ratooning capacity of most existing cultivars, resulting in insufficient tiller density per hectare. To address this challenge, the breeding and selection of rice varieties with enhanced ratooning ability have become pivotal strategies for improving yield stability and realizing the full potential of ratoon rice systems.

Ratooning rice is a cultivation system in which the stubble of the first crop is retained after harvest, enabling dormant axillary buds to sprout and develop into new panicles, thereby producing a second yield [5,6]. The ratooning ability denotes the potential of these dormant buds on the stubble to sprout under favorable conditions following the main harvest, develop into new tillers, and subsequently advance to heading and maturity, ultimately realizing a second crop [7]. A growing body of evidence indicates that the regeneration capacity of ratoon rice is profoundly influenced by agronomic factors, including the timing of the main crop harvest [8], stubble height [9], and the management of water and fertilizer during the ratoon season [10,11].In addition to these management practices, regeneration potential is also modulated by physiological processes such as root activity and carbohydrate accumulation capacity [12]. Among these physiological traits, the viability and sprouting potential of axillary buds are particularly critical determinants of regeneration, as they directly shape tillering capacity and final yield. At the molecular level, the outgrowth of axillary buds is regulated by genes responsible for axillary meristem initiation. Previous studies identified *MOC1*(*MONOCULM1*), *LAX1*(*LAX PANICLE 1*), and *LAX2*(*LAX PANICLE 2*) as promoters of tiller formation in rice, while, *HTD1*(*HIGH-TILLERING DWARF 1*) and *TAD1*(*TILLERING and DWARF 1*) act as inhibitors [13]. These genes function through distinct pathways that control meristem establishment, signal transduction, and strigolactone (SL) biosynthesis, thereby collectively determining whether axillary buds remain dormant or initiate growth. The regeneration of rice axillary buds is further controlled by a complex network of transcription factors that integrate multiple hormone signaling pathways. Under phosphorus deficiency, *NSP1*(*NODULATION SIGNALING PATHWAY1*) and *NSP2*(*NODULATION SIGNALING PATHWAY1*) enhance strigolactone (SL) biosynthesis in rice roots, thereby suppressing axillary bud growth through SL signaling [14]. Recent findings reveal that *OsSHI1* (*SHORT INTERNODES1*) directly modulates genes involved in auxin (IAA) and brassinosteroid (BR) biosynthesis [15]. By modulating the transcriptional landscape of phytohormone pathways, these genes link hormonal homeostasis with axillary meristem activity.

The growth of axillary buds is orchestrated by multiple plant hormones, including IAA, cytokinin (CK), SL, abscisic acid (ABA), and gibberellin (GA). These hormones do not act independently; instead, they form an integrated regulatory network that collectively determines bud dormancy, initiation, and elongation [16,17]. Among them, IAA and SL function as major inhibitory signals restricting axillary bud outgrowth [18,19]. IAA, synthesized in the apical bud, is transported basipetally and accumulates in the stem, where it indirectly suppresses axillary bud growth by modulating the biosynthesis and distribution of SL [20,21] and CK [22]. SL, synthesized in the roots and stem base, moves upward to axillary buds and amplifies IAA-mediated inhibition by promoting the expression of the transcription factor BRC1 [23]. In contrast, CK acts as the primary growth-promoting hormone for axillary buds. It is mainly synthesized in the roots and translocated via the xylem to axillary buds, where it promotes cell division and expansion [24]. An antagonistic interaction exists between IAA and CK: IAA reduces CK accumulation to inhibit bud outgrowth, whereas CK counteracts IAA-mediated inhibition to stimulate bud activation [22]. ABA also plays a central role in maintaining bud dormancy, particularly under environmental stress [25]. Stress conditions induce ABA accumulation in dormant buds, reinforcing growth suppression, whereas decapitation depletes ABA and releases bud dormancy. Emerging evidence indicates that ABA acts synergistically with SL to strengthen inhibition, while CK antagonizes ABA signaling to promote bud outgrowth [26]. These interactions reveal a tightly coordinated hormonal network that determines the developmental fate of axillary buds. Together, these interactions reveal a highly integrated hormonal network that precisely regulates the developmental fate of axillary buds.

In our preliminary study, we systematically evaluated the regeneration capacity of 130 rice varieties and identified a set of cultivars with high and low regeneration ability. Through large-scale transcriptome sequencing, we identified a differentially expressed gene, O*sPM19L*, whose expression was markedly higher in high-regeneration cultivars than in low-regeneration ones. Moreover, in high-regeneration varieties, *OsPM19L* expression sharply declined following the first-season harvest. Previous studies have reported that *OsPM19L* encodes a plasma membrane–localized protein that facilitates the cellular influx of ABA. In the ABA signaling pathway, transporter proteins mediate both intercellular and intracellular movement of ABA. These transport processes not only establish localized hormone gradients that trigger rapid stress responses but also restore hormonal homeostasis to terminate signaling, thereby ensuring precise regulation of plant adaptation and developmental processes [27]. As a key growth-inhibiting phytohormone, ABA has been established as a core negative regulator controlling bud dormancy and outgrowth. The accumulation of ABA in the nodal regions of rice significantly suppresses axillary bud outgrowth, thereby reducing regeneration capacity [28]. In this study, we generated *OsPM19L* mutant line (*ospm19l*) and overexpression line (*OsPM19L-OE*) through genetic transformation (Supplemental Figure S1). Multiple ratooning-related traits were quantitatively analyzed, and phytohormone levels in plant tissues were measured to further elucidate the role of *OsPM19L* and its regulatory mechanism in rice axillary bud outgrowth.

## 2. Materials and Methods

### 2.1. Construction and Validation of Transgenic Materials

Materials used in this study were of the conventional japonica cultivar Zhonghua 11 (ZH11) background. For the generation of *OsPM19L* overexpression lines, the full-length coding sequence of *OsPM19L* was cloned into a plant expression vector under the control of the CaMV 35S promoter. *ospm19l* mutants were generated using CRISPR/Cas-mediated genome editing. Briefly, target-specific gRNA sequences were designed and inserted into a CRISPR/Cas gene editing vector.

The resulting recombinant vectors were introduced into ZH11 rice via Agrobacterium tumefaciens-mediated transformation of mature embryo-derived calli. Transgenic plants were selected based on antibiotic resistance, and integration and expression of *OsPM19L* were verified by sequencing and qRT–PCR. Stable overexpression and gene-edited lines were obtained after successive generations of selfing. Primer sequences, vector backbones, and validation data for positive lines are provided in the Supplementary Materials.

### 2.2. Seedling Cultivation and Field Management

Rice seeds were soaked in water for 2 days, and floating or non-viable seeds were removed. The remaining seeds were placed on moist germination paper to germinate. Germinated seedlings were transferred to 96-well germination trays and grown in a controlled growth chamber at 28 °C with a 14 h/10 h (day/night) photoperiod. Seedlings at the two-leaf stage were transplanted into pots or the field for cultivation.

Field experiments were conducted at the experimental station of Hunan Agricultural University, Changsha, Hunan Province, China (latitude 28° 18′, longitude 113° 03′). A randomized complete block design was used, with three biological replicate plots per treatment. Each plot measured 5 m × 5 m, with row spacing of 20 cm and plant spacing of 15 cm. Basal fertilizer was applied at a rate of N:P:K = 45:40:40 kg/ha, and additional fertilizer was applied at the tillering stage, heading stage, and five days after harvest. Irrigation was applied as needed to maintain adequate soil moisture. At harvest, a stubble height of 30 cm was maintained.

### 2.3. Subcellular Localization

The open reading frame of *OsPM19L* was cloned into the pRI101-EGFP vector at the Sal I and Kpn I restriction sites by homologous recombination, generating the 35Spro:EGFP-*OsPM19L* fusion construct. Agrobacterium tumefaciens strains harboring 35Spro:EGFP-*OsPM19L* or 35Spro:EGFP (control) were independently infiltrated into the abaxial sides of leaves of 4–6-week-old *Nicotiana benthamiana* plants. For plasma membrane localization reference, a construct expressing an mCherry-tagged plasma membrane marker was co-infiltrated as a membrane localization marker. After infiltration, plants were maintained under standard light conditions for 48 h. Fluorescence signals in the infiltrated leaf areas were examined using a confocal laser scanning microscope (Leica, Wetzlar, Germany). EGFP was excited with a 488 nm argon laser, and the emitted fluorescence was collected between 500 and 530 nm. Leaves expressing free EGFP were used as negative controls to exclude nonspecific fluorescence and background signals.

### 2.4. RNA Extraction and qRT-PCR Analyses

Total RNA was extracted from various rice tissues using TRIzol reagent (CWBIO, Taizhou, China), and first-strand cDNA was synthesized using a reverse transcription kit according to the manufacturer’s instructions. Quantitative real-time PCR (qRT-PCR) was performed on a CFX96 Real-Time PCR Detection System (Bio-Rad, Hercules, CA, USA) using ArtiCanCEO SYBR qPCR Mix (Tsingke, Beijing, China) following the supplier’s protocol. For the qRT–PCR analysis, gene expression levels were normalized using OsActin as the internal control, and relative expression levels were calculated using the $2^{-\Delta \Delta Ct}$ method. The gene IDs used in this study, together with the corresponding primer sequences, are listed in the Supplementary Materials.

### 2.5. Promoter Sequence Analysis

The cis-acting regulatory elements in the *OsPM19L* promoter, isolated from ZH11 genomic DNA, were identified using the PlantCARE (https://bioinformatics.psb.ugent.be/webtools/plantcare/html/, accessed on 10 December 2025) online tool. The distribution of these elements was subsequently visualized to characterize potential regulatory motifs.

### 2.6. Phytohormone Treatments and OsPM19L Expression

To analyze the effects of different phytohormones on *OsPM19L* expression, 14-day-old ZH11 seedlings were treated with hormones. All hormones were purchased from Coolaber (Beijing, China), with IAA and KT dissolved in sterile water and GA and ABA dissolved in dimethyl sulfoxide (DMSO). Seedlings were transferred to liquid media containing ABA, GA, KT, or IAA at a concentration of 5 μM. Leaf samples were collected 24 h after treatment for *OsPM19L* expression analysis.In addition, the expression of *OsPM19L* was examined under different ABA concentrations (5, 10, and 25 μM) and at different treatment durations (0, 6, and 24 h).

### 2.7. GUS Histochemical Staining and Quantitative Analysis

The *OsPM19L* promoter fragment was cloned into the pCAMBIA3301-GUS vector to generate the *OsPM19Lpro::GUS* reporter construct and introduced into *Agrobacterium tumefaciens*. The recombinant Agrobacterium suspension was infiltrated into leaves of 4–6-week-old *Nicotiana benthamiana* plants. After infiltration, plants were incubated under light conditions for 48 h. The infiltrated leaves were then treated with different concentrations of ABA (0, 0.5, 1, and 10 μM) for 24 h. Subsequently, the leaves were immersed in GUS staining solution containing 1 μM X-Gluc, 100 μM sodium phosphate buffer (pH 7.2), 10 μM EDTA, 0.5 μM K_3_[Fe(CN)_6_], 0.5 μM K_4_[Fe(CN)_6_], and 0.1% Triton X-100, and incubated at 37 °C in the dark for 12 h. After staining, the tissues were decolorized with 95% ethanol in a 70 °C water bath to visualize blue GUS staining.

For quantitative analysis, 100 mg of fresh leaf tissue was ground in liquid nitrogen, and total proteins were extracted using GUS protein extraction buffer. Protein concentration was determined by the Bradford method. Fluorescence intensity was measured using a microplate reader at an excitation wavelength of 365 nm and an emission wavelength of 455 nm. A standard curve was generated using different concentrations of 4-methylumbelliferone (4-MU), with a correlation coefficient of R^2^ > 0.995. GUS activity was defined as the amount of 4-MU produced per minute per microgram of protein (pmol MU·min^−1^ · μg^−1^ protein).

### 2.8. Seed Germination and Seedling Growth Assays

For seed germination and seedling growth assays, sterilized mature seeds of ZH11, *ospm19l*, and *OsPM19L-OE* lines were placed on filter papers saturated with sterile water (control), 2.5 μM ABA, 5 μM ABA, or 10 μM ABA. The seeds were incubated in a growth chamber under controlled conditions (28 °C, 14 h light/10 h dark). Germination rates were recorded every 24 h, with three replicates of 50 seeds per treatment. After germination, seedlings were transferred to hydroponic solutions containing the corresponding ABA concentrations and grown for an additional 14 days, after which plant length was measured.

Seeds of all transgenic lines were from selfed, stably inherited T2 generations, harvested simultaneously with ZH11 to ensure comparable physiological status.

### 2.9. Evaluation of Ratoon Phenotype

Wild-type plants, *ospm19l*, and *OsPM19L-OE* were grown at a standard natural field experimental site under uniform cultivation and management practices. At the maturity stage of the main season, the number of effective panicles (MPN) per plant was recorded, with at least 15 plants examined for each genotype. At maturity of the ratoon season, the effective panicles number in ratoon season (RPN) were quantified using the same procedure. To assess ratooning ability, the ratio of ratoon to main-season panicle number (RMP) was calculated using the following formula:

$$\mathrm{RMP}=\frac{\mathrm{effective}\;\mathrm{panicles}\;\mathrm{number}\;\mathrm{in}\;\mathrm{ratoon}\;\mathrm{season}\;\left(\mathrm{RPN}\right)}{\mathrm{effective}\;\mathrm{panicles}\;\mathrm{number}\;\mathrm{in}\;\mathrm{main}\;\mathrm{season}\;\left(\mathrm{MPN}\right)}$$

The RMP index was used to compare the extent of panicle number changes from the main season to the ratoon season across genotypes, thereby reflecting their differences in ratooning capacity.

The axillary buds were measured at 0 and 3 days after harvesting.

### 2.10. Phytohormone Determinations

The extraction and quantification of phytohormones, including Z, ZR, IAA, GAs, and ABA, were performed following previously described methods, using a liquid chromatography–tandem mass spectrometry (LC–MS/MS) system for detection. Fresh axillary buds samples (200 mg) were finely ground in liquid nitrogen and extracted with 1 mL of a mixed solvent containing 75% methanol, 20% water, and 5% formic acid. The mixture was vortexed thoroughly and incubated at 4 °C for 10 h. After extraction, the samples were centrifuged at 10,000× *g* for 10 min at 4 °C, and the supernatant was collected and vacuum-dried. The residue was re-dissolved in 100 μL of ultrapure water, centrifuged for 5 min, and 5 μL of the supernatant was injected into the LC–MS system for analysis. Chromatographic separation was carried out on an ACQUITY UPLC HSS T3 column (2.1 mm × 100 mm, Waters) maintained at 40 °C. The mobile phase consisted of solvent A (0.1% formic acid in water) and solvent B (acetonitrile), with a gradient elution program summarized as “time (min)/A (%)/B (%)”: 0/90/10, 6/83/17, 12/75/25, 22/90/10.

The mass spectrometry system employed electrospray ionization (ESI) and analyzed different hormones under multiple reaction monitoring (MRM) mode. ZR was measured with a collision energy of 19 eV and a mass-to-charge ratio (*m*/*z*) of 352.2/220.1, while Z used a collision energy of 25 eV and *m*/*z* 225/137.1. GA3 was analyzed with a collision energy of 20 eV and *m*/*z* 431.3/386.2, and GA4 with a collision energy of 28 eV and *m*/*z* 417.4/372.2. IAA was detected using a collision energy of 18 eV and *m*/*z* 215.2/260.2, and ABA with a collision energy of 18 eV and *m*/*z* 354.2/309.4.

In this study, quantification was performed using the external standard method. Gradient concentrations of authentic standards were prepared to establish calibration curves for each target hormone. Method validation demonstrated good linearity for all hormones within the tested concentration ranges, with correlation coefficients (R^2^) greater than 0.999. Hormone levels are now clearly presented as ng/g fresh weight (FW), and replicate measurements were conducted to ensure the reproducibility and reliability of the experimental data.

### 2.11. Preparation and Observation of Axillary Bud Paraffin Sections

Axillary buds were collected from rice at the yellow ripening stage (pre-harvest) and 72 h post-harvest. Buds at the second internode were quickly excised and immediately fixed in 50% FAA (Formalin-Aceto-Alcohol) at 4 °C for 24 h. After fixation, samples were rinsed with 50% ethanol and dehydrated through a graded ethanol series: 50% for 45 min, 70% for 1 h, 85% for 1 h, 95% I for 1 h, and 100% II. Dehydrated tissues were then cleared and infiltrated with paraffin in the following sequence: ethanol:xylene (1:1) for 10 min, xylene for 8 min × 2, paraffin I for 1 h, paraffin II for 1 h, and paraffin III for 1 h. Samples were embedded in paraffin, cooled on a freezing platform, and sectioned at 3–5 μM using a microtome. Sections were transferred onto slides, flattened, and dried. Sections were deparaffinized, rehydrated, and stained with hematoxylin and eosin for 3–5 min. After dehydration and clearing with ethanol and xylene, slides were air-dried and mounted with neutral resin. Morphological observations and imaging were conducted using a wide-field optical microscope (Axio Imager 2, ZEISS, Jena, Germany).

## 3. Results

### 3.1. Spatiotemporal Expression Pattern of OsPM19L and Its Response to Harvest Operations

**Figure 1:**
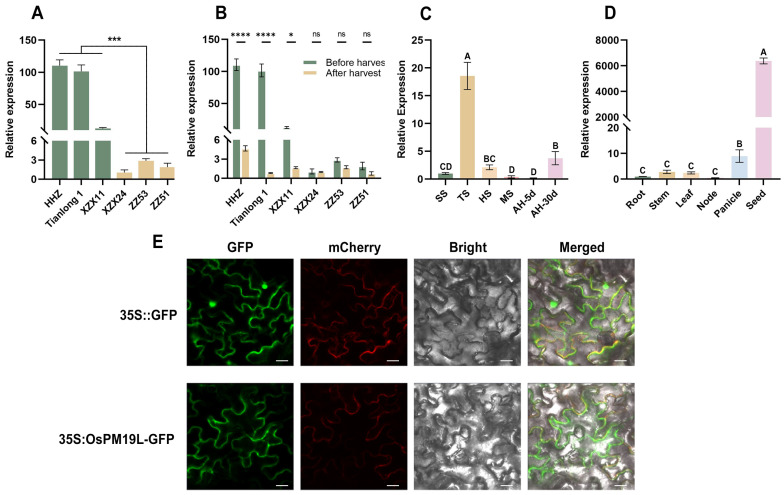
Expression characteristics of *OsPM19L* in rice. (**A**) Relative expression levels of *OsPM19L* in strong-regeneration cultivars (HHZ, Tianlong 1, ZX11) and weak-regeneration cultivars (ZZ24, ZZ53, ZZ51). (**B**) Relative expression of OsPM19L before and after harvest in different rice cultivars. (**C**) Expression pattern of *OsPM19L* at different developmental stages of wild-type rice. SS, seedling stage; TS, tillering stage; HS, heading stage; MS, maturity stage; AH-5d, 5 days after harvest; AH-30d, 30 days after harvest. (**D**) Expression pattern of *OsPM19L* in different tissues of wild-type rice. (**E**) Subcellular localization of *OsPM19L* in Nicotiana benthamiana. GFP, green fluorescence; mCherry, plasma membrane marker; Bright, bright-field images; Merged, overlay of all channels. Bar = 20 μm. In Figure 1, data are presented as means ± SD from three independent biological replicates (n = 3), with each biological replicate including three technical replicates. Statistical analysis was performed using one-way ANOVA, followed by Tukey’s HSD post-hoc test to assess differences among multiple groups. Normality and homogeneity of variances were confirmed prior to analysis. Different letters indicate statistically significant differences at *p* < 0.01 based on Tukey’s test. Significant differences between two specific groups were further evaluated using Student’s *t*-test: *p* > 0.05 (ns), *p* < 0.05 (*), *p* < 0.001 (***), *p* < 0.0001 (****).

Transcriptome analysis of axillary buds from rice cultivars with contrasting regeneration capacities revealed that *OsPM19L* was highly expressed in strong-regeneration cultivars(HHZ, Tianlong1, XZX11) but exhibited low expression in weak-regeneration ones(XZX24, ZZ51, ZZ53). Based on these transcriptomic results, we further performed qRT–PCR validation and obtained a consistent expression pattern (Figure 1A). In several strong-regeneration varieties, its expression was markedly downregulated 3 days after harvest, indicating a rapid response to harvest-induced signals (Figure 1B). Sequence analysis showed that the *OsPM19L* coding sequence is 522 bp in length and encodes a 173-amino-acid protein containing a single AWPM-19 domain. Phylogenetic analysis revealed high sequence similarity between *OsPM19L* and other AWPM-19 domain-containing proteins from various plant species (Supplemental Figure S2), and subcellular localization assays confirmed its presence in the plasma membrane (Figure 1E). The spatial and temporal expression of genes often reflects their physiological functions. qRT-PCR analysis demonstrated that *OsPM19L* was expressed in multiple rice tissues, including roots, stems, leaves, nodes, panicles, and seeds, with particularly high levels in panicles and seeds (Figure 1C). During plant development, *OsPM19L* expression peaked at the tillering stage, decreased during grain maturity, and further declined after harvest before gradually recovering 30 days postharvest (Figure 1D). These results suggest that *OsPM19L* may play a regulatory role in rice tillering and axillary bud growth following harvest.

### 3.2. OsPM19L Is Strongly Induced by ABA and Acts as a Positive Regulator in ABA Signaling

**Figure 2:**
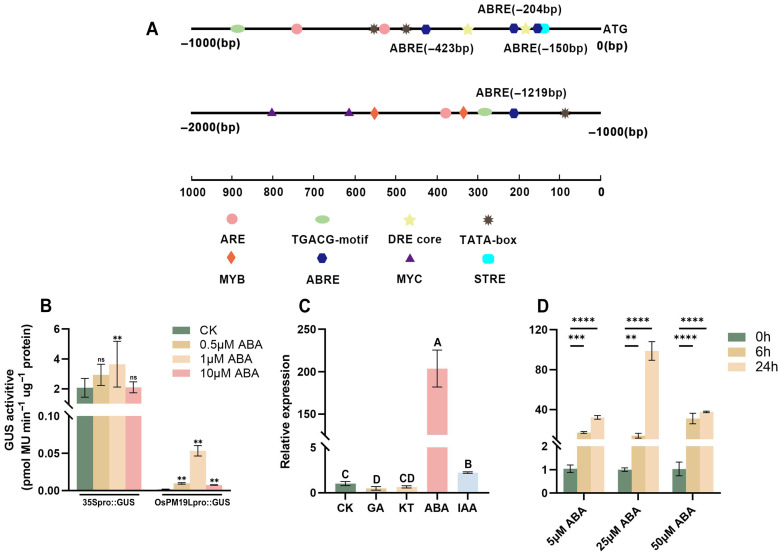
Functional characterization of *OsPM19L* in response to hormones. (**A**) Cis-acting elements identified in the 2-kb upstream promoter region of *OsPM19L*. (**B**) Transient expression of OsPM19Lpro:GUS in *Nicotiana benthamiana* leaves under different ABA concentrations. (**C**) Relative expression levels of *OsPM19L* under different phytohormone treatments. (**D**) Relative expression levels of *OsPM19L* in WT seedlings treated with different concentrations of ABA for various durations. In Figure 2, data are presented as means ± SD ((**B**–**D**): n = 3). Statistical analysis was performed using one-way ANOVA, followed by Tukey’s HSD post-hoc test to assess differences among multiple groups. Normality and homogeneity of variances were confirmed prior to analysis. Different letters indicate statistically significant differences at *p* < 0.01 based on Tukey’s test. Significant differences between two specific groups were further evaluated using Student’s *t*-test: *p* > 0.05 (ns), *p*< 0.01 (**), *p* < 0.001 (***), *p* < 0.0001 (****).

Promoter analysis of approximately 2000 bp upstream of the *OsPM19L* coding sequence revealed the presence of multiple hormone-responsive cis-elements, including ABRE motifs that are commonly found in ABA-inducible genes and can interact with bZIP transcription factors to regulate gene expression (Figure 2A). Transient expression assays in tobacco further confirmed that the *OsPM19L* promoter was activated by ABA in a dose-dependent manner, with GUS activity increasing significantly under ABA treatment (Figure 2B, Supplemental Figure S3).

**Figure 3:**
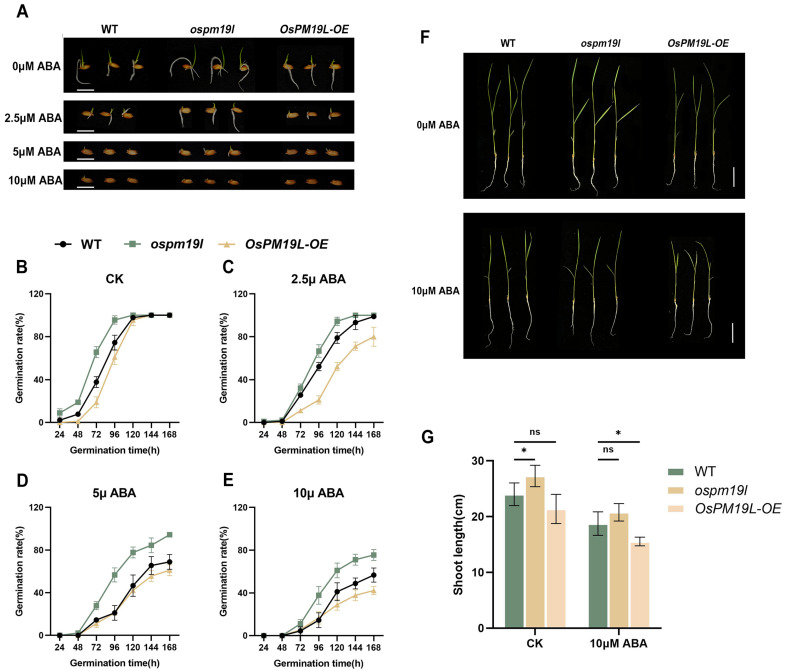
Functional characterization of *OsPM19L* in response to ABA. (**A**) Seed germination phenotypes of WT and *OsPM19L* transgenic lines under different ABA concentrations. Bar = 1 cm. (**B**–**E**) Germination rate curves of WT and *OsPM19L* transgenic lines under different ABA concentrations. (**F**) Seedling growth phenotypes of WT and *OsPM19L* transgenic lines after 14 days of treatment under control and ABA conditions. Bar = 5 cm. (**G**) Comparison of shoot length among genotypes under different ABA concentrations.In Figure 3, data are presented as means ± SD ((**B**–**E**): n = 50, (**G**): n = 10). Statistical analysis was performed using one-way ANOVA, followed by Tukey’s HSD post-hoc test to assess differences among multiple groups. Normality and homogeneity of variances were confirmed prior to analysis. Significant differences between two specific groups were further evaluated using Student’s *t*-test: *p* > 0.05 (ns), *p* < 0.05 (*).

To examine whether *OsPM19L* is responsive to plant hormones, 14-day-old wild-type rice seedlings were treated with various hormones.Leaf samples for expression analysis were collected at 24 h after treatment. The results showed that *OsPM19L* was strongly induced by ABA, with expression peaking 24 h after 25 μM ABA treatment (Figure 2C,D). In seed germination assays under different ABA concentrations, *ospm19l* seeds germinated faster than wild-type under control conditions, while *OsPM19L-OE* seeds germinated more slowly. Exogenous ABA markedly inhibited germination in all genotypes, and as ABA concentration increased, *OsPM19L-OE* exhibited higher ABA sensitivity, whereas *ospm19l* showed reduced sensitivity (Figure 3A–E). Because ABA is known to inhibit seedling growth, we compared shoot length among genotypes under both normal and ABA-treated conditions. Under control conditions, *ospm19l* plants displayed significantly longer shoots than wild-type, whereas *OsPM19L-OE* showed no significant difference. After 14 days of growth in 10 μM ABA, *OsPM19L-OE* plants were significantly shorter than wild-type, while *ospm19l* plants were slightly longer but not significantly different (Figure 3F,G). Taken together, these results demonstrate that *OsPM19L* acts as a positive regulator in ABA signaling, contributing to ABA-mediated inhibition of seed germination and seedling growth in rice.

### 3.3. OsPM19L Regulates Tillering in the Main Season and Early Axillary Bud Outgrowth in the Ratoon Season

**Figure 4:**
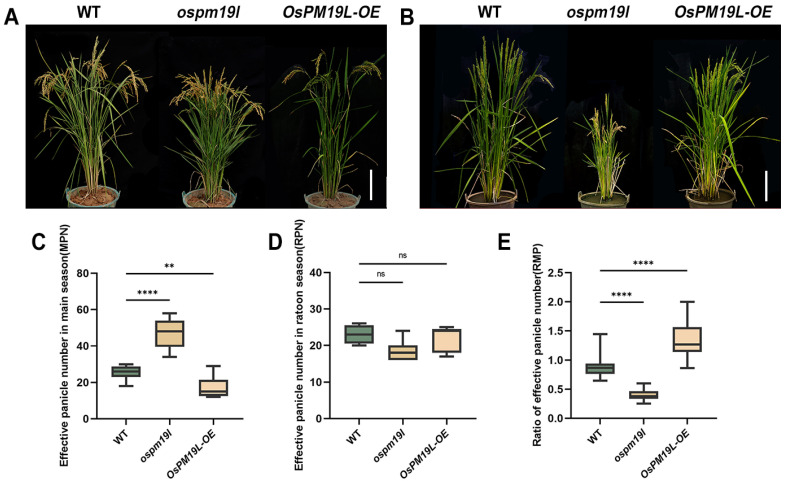
Functional characterization of *OsPM19L* in tiller formation during the main and ratoon seasons. (**A**) Phenotypes of WT and *OsPM19L* transgenic lines plants at the heading stage of the maturity stage. Bar = 20 cm. (**B**) Phenotypes of WT and O*sPM19L* transgenic lines in the ratoon season. Bar = 20 cm. (**C**) Effective panicle number in main season, MPN. (**D**) Effective panicle number in ratoon season, RPN. (**E**) Ratio of effective panicle number, RMP. In Figure 4, data are presented as means ± SD ((**C**–**E**): n = 15). Statistical analysis was performed using one-way ANOVA, followed by Tukey’s HSD post-hoc test to assess differences among multiple groups. Normality and homogeneity of variances were confirmed prior to analysis. Significant differences between two specific groups were further evaluated using Student’s *t*-test: *p* > 0.05 (ns), *p*< 0.01 (**), *p* < 0.0001 (****).

To investigate the phenotypic effects of *OsPM19L* under natural field conditions, the wild-type, *ospm19l*, and *OsPM19L-OE* overexpression lines were grown and evaluated (Figure 4A,B). Prior to the main-season harvest, the *ospm19l* plants exhibited a significantly higher number of effective panicles than the wild type, whereas O*sPM19L-OE* plants showed markedly fewer effective panicles (Figure 4C). When plants reached maturity in the ratoon season (approximately two months after harvest), no significant difference in the absolute number of effective panicles was observed among genotypes (Figure 4D). However, analysis of the RMP (ratio of ratoon to main panicle number) revealed that *ospm19l* had a significantly lower index than the wild type, while *OsPM19L-OE* displayed a higher one (Figure 4E). This indicates that *OsPM19L-OE* plants possess a stronger intrinsic ratooning ability relative to their main crop performance.

**Figure 5:**
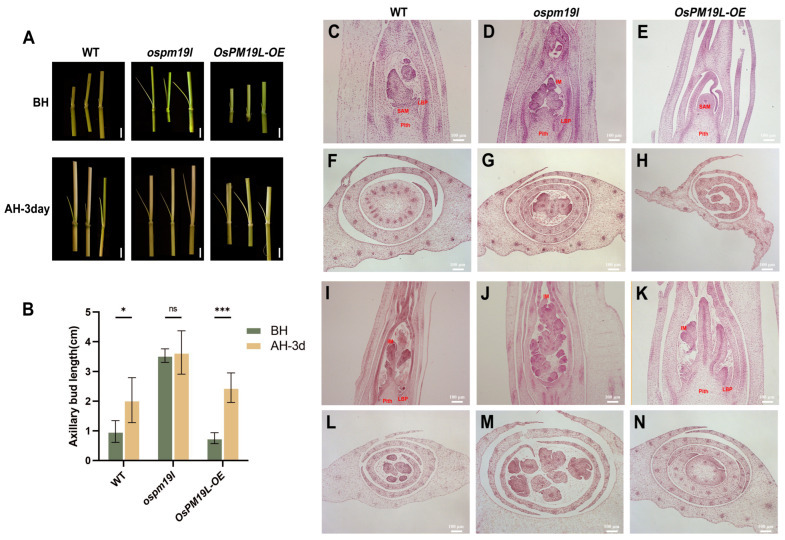
Functional characterization of *OsPM19L* in axillary bud development during the main and ratoon seasons. (**A**) Axillary bud morphology of WT and *OsPM19L* transgenic lines before harvest and after harvest 3 days. Bar = 1 cm. (**B**) Length of axillary buds before harvest and after harvest 3 days. (**C**–**H**) Paraffin sections of axillary buds before harvest. (**C**–**E**), Longitudinal sections; (**F**–**H**), cross sections. Bar = 100 μM. (**I**–**N**) Paraffin sections of axillary buds three days after harvest. (**I**–**K**), Longitudinal sections; (**L**–**N**), cross sections. Bar = 100 μM. SAM: Shoot apical meristem; LBP: Lateral bud primordium; IM: Inflorescence meristem. Figure 5, data are presented as means ± SD ((**B**): n = 5). Statistical analysis was performed using one-way ANOVA, followed by Tukey’s HSD post-hoc test to assess differences among multiple groups. Normality and homogeneity of variances were confirmed prior to analysis. Significant differences between two specific groups were further evaluated using Student’s *t*-test: *p* > 0.05 (ns), *p* < 0.05 (*), *p* < 0.001 (***).

These results indicate that *OsPM19L* negatively regulates tiller formation during the main season. To further investigate its role in axillary bud outgrowth and early growth during the ratoon season, we conducted a time-course analysis of bud length across different genotypes. Three days after harvest, the axillary buds of the wild type exhibited a rapid increase in length, indicating prompt activation of regeneration. In contrast, *OsPM19L-OE* buds elongated more vigorously, showing enhanced regenerative potential, whereas *ospm19l* buds, though longer in absolute length, displayed a delayed growth response (Figure 5A,B).

To elucidate the anatomical basis of these phenotypic differences, paraffin sectioning was performed at multiple developmental stages. Before harvest, axillary buds of *ospm19l* had already developed visible leaf primordia and early inflorescence structures, suggesting premature differentiation. Conversely, *OsPM19L-OE* buds were smaller but contained larger and denser shoot apical meristems, with compact, actively dividing cells indicative of a high readiness for regrowth (Figure 5C–H). Three days after harvest, all genotypes showed increased bud differentiation, but *OsPM19L-OE* buds displayed accelerated formation of leaf and inflorescence primordia (Figure 5I–N). These anatomical observations were consistent with the bud elongation measurements, demonstrating that *OsPM19L-OE* buds have a higher initial growth rate during early ratoon development. Collectively, these results suggest that *OsPM19L* acts as a negative regulator of tiller formation during the main season but functions as a positive regulator of dormant axillary bud outgrowth during the early ratoon stage.

### 3.4. OsPM19L Coordinates Phytohormone Signaling to Promote Axillary Bud Outgrowth

**Figure 6:**
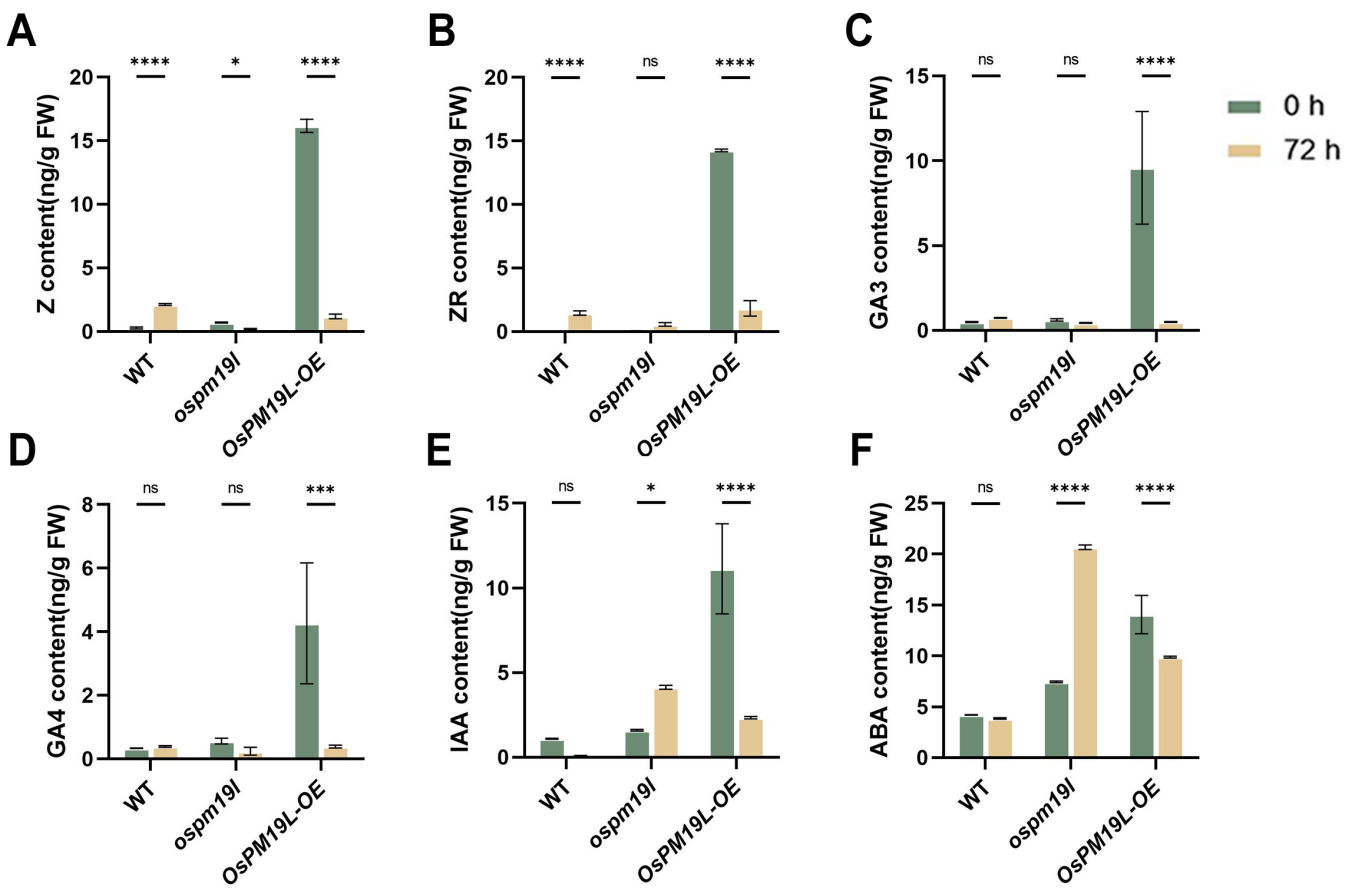
Hormone contents in axillary buds of wild-type and *OsPM19L* transgenic lines before harvest and three days after harvest. (**A**) Z, zeatin; (**B**) ZR, zeatin riboside; (**C**) GA3, gibberellins 3; (**D**) GA4, gibberellins 4; (**E**) IAA, indole-3-acetic acid; (**F**) ABA, abscisic acid. In Figure 6, data are presented as means ± SD (n = 3). Statistical analysis was performed using one-way ANOVA, followed by Tukey’s HSD post-hoc test to assess differences among multiple groups. Normality and homogeneity of variances were confirmed prior to analysis. Significant differences between two specific groups were further evaluated using Student’s *t*-test: *p* > 0.05 (ns), *p* < 0.05 (*), *p* < 0.001 (***), *p* < 0.0001 (****).

After rice harvest, dormant axillary buds break dormancy and initiate regeneration, a process finely regulated by multiple phytohormones. To investigate hormonal regulation during bud outgrowth, we measured hormone levels in axillary buds immediately before harvest and three days post-harvest. Zeatin (Z) is the biologically active form of cytokinin that directly stimulates cell division, whereas zeatin riboside (ZR) serves as a storage or transport form that can be rapidly converted into Z when needed. The axillary buds of *OsPM19L-OE* plants exhibited higher CK and GA levels before harvest, which declined three days after harvest. This pattern suggests that the stored CK and GA may be rapidly mobilized to initiate bud dormancy release and support early axillary bud growth (Figure 6A–D). During this process, CKs primarily activate meristems and initiate cell division, whereas GAs mainly promote cell elongation; together, they synergistically drive dormancy release and early growth. *OsPM19L-OE* buds maintained high levels of both CKs and GAs, facilitating efficient initiation of cell division and elongation and providing strong regenerative potential. IAA functions mainly to maintain apical dominance by inhibiting axillary bud outgrowth. After harvest, IAA sources are removed, and IAA levels decreased significantly in wild-type and *OsPM19L-OE* buds, releasing dormancy. In contrast, *ospm19l* buds exhibited elevated IAA, indicating that residual IAA was not effectively cleared, thereby suppressing bud growth(Figure 6E). ABA, a key stress-responsive hormone that maintains dormancy and inhibits growth, must decline for dormancy release. Compared to wild-type, ABA levels in *OsPM19L-OE* buds dropped markedly after harvest. By contrast, ABA levels in *ospm19l* buds rised, preventing effective regeneration(Figure 6F). Based on the combined hormone profiling results, we propose that *OsPM19L* may contribute to axillary bud dormancy release and early growth by coordinating relatively high CK and GA levels while concomitantly maintaining lower IAA and ABA levels.

**Figure 7:**
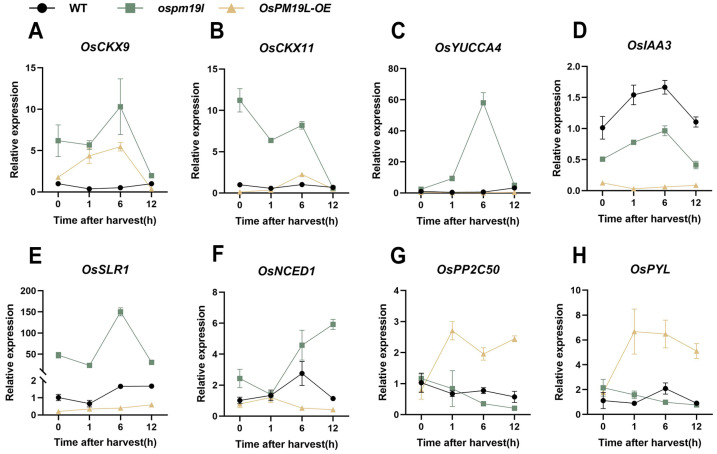
Expression of hormone-related genes in axillary buds of different genotypes before and after harvest. (**A**) *OsCKX9*; (**B**) *OsCKX11*; (**C**) *OsYUCCA4*; (**D**) *OsIAA3*; (**E**) *OsSLR1*; (**F**) *OsNCED1*; (**G**) *OsPP2C50*; (**H**) *OsPYL*. In Figure 7, data are presented as means ± SD (n = 3). Statistical analysis was performed using one-way ANOVA, followed by Tukey’s HSD post-hoc test to assess differences among multiple groups. Normality and homogeneity of variances were confirmed prior to analysis.

These results indicate that distinct hormone signaling patterns exist among genotypes during ratoon bud outgrowth. To further characterize these differences at the transcriptional level, we examined the expression of key genes in hormone pathways. The cytokinin oxidase gene *OsCKX9*, which catalyzes CK degradation, showed a similar temporal pattern in *ospm19l* and *OsPM19L-OE*, increasing initially and then declining; however, its expression remained consistently higher in *ospm19l*. Similarly, *OsCKX11* expression was higher in *ospm19l* throughout the time course (Figure 7A,B). *OsYUCCA4*, a key gene for IAA biosynthesis, peaked at 6 h post-harvest in *ospm19l* buds (Figure 7C). Moreover, the expression level of the IAA negative regulator OsIAA3 was higher in *ospm19l* than in *OsPM19L-OE* (Figure 7D). The GA signaling repressor *OsSLR1* showed an increasing trend after harvest, with a more pronounced rise in *ospm19l* (Figure 7E). For ABA biosynthesis, *OsNCED1* expression was high in *ospm19l* and further increased over time, whereas in *OsPM19L-OE*, it remained low and declined. Moreover, the ABA receptor *OsPYL3* and the negative regulator of ABA signaling *OsPP2C50* increased sharply in *OsPM19L-OE* after harvest but decreased in o*spm19l* (Figure 7F–H). These transcriptional patterns generally consistent with the observed hormone contents, explaining the post-harvest decline of ABA in *OsPM19L-OE* buds and its accumulation in *ospm19l* buds.

## 4. Discussion

The AWPM-19 family has been characterized as a group of highly conserved hydrophobic proteins that generally function as positive regulators in enhancing plant stress tolerance. However, their roles during reproductive development appear to be more complex. In Arabidopsis thaliana, AtPM19L1 acts as a negative regulator [29], whereas its homologs in rice and wheat exhibit positive regulatory functions [30,31]. Previous studies have demonstrated that *OsPM19L* encodes an ABA influx protein, and increased ABA concentration enhances stress resistance in plants [32]. ABA transporters play key roles in regulating stomatal movement, ABA signal transduction, and drought tolerance in plants. In this study, OsPM19L expression displayed distinct spatiotemporal specificity, responding to harvest signals and being strongly induced by ABA. Phenotypic differences in germination and growth between the *ospm19l* and *OsPM19L-OE* overexpression lines under exogenous ABA treatment further confirmed that *OsPM19L* functions as a positive regulator in the ABA signaling pathway (Figure 1, Figure 2 and Figure 3).

Ratoon rice provides an efficient approach to improve rice productivity and resource use efficiency, and its yield potential largely depends on the regeneration ability of axillary buds. Axillary buds that break dormancy can grow into new ears, allowing for a second harvest. Genetic and physiological analyses revealed that *OsPM19L* negatively regulates tillering during the main season but promotes axillary bud activation and regeneration during the ratoon season. These observations suggest that *OsPM19L* is likely to perform distinct functions at different developmental stages, which may be approximately associated with the plant’s physiological status and environmental context. The main-season harvest represents a considerable wounding and stress event, potentially triggering rapid changes in endogenous hormone levels, and *OsPM19L* appears to sense or participate in modulating these hormonal fluctuations. Furthermore, between the main and ratoon seasons, the proteins potentially interacting with *OsPM19L* and the transcription factors regulating its expression may differ, which could lead to divergent expression patterns and functional outcomes of *OsPM19L* (Figure 4). Unlike conventional ratooning evaluations, this study quantitatively measured early axillary bud growth rates and combined them with cytological analyses to elucidate the structural basis of regeneration (Figure 5). Anatomical observations at early post-harvest stages showed that *OsPM19L-OE* buds possessed compact and actively dividing apical meristems, indicating a primed state for outgrowth, whereas *ospm19l* buds exhibited premature differentiation with visible leaf and inflorescence primordia. This structural divergence explains why *ospm19l* plants produced more panicles in the main season but exhibited reduced regeneration efficiency—premature differentiation favors reproductive allocation in the main crop but compromises regenerative potential.

After initiation, rice axillary buds may either directly differentiate into lateral branches to form new tillers or enter a transitional dormant state [33]. The transition from dormancy to outgrowth is coordinately regulated by multiple phytohormones, including ABA, GA, and IAA. In recent years, substantial progress has been made in elucidating the hormonal regulation of plant branching. It has been well established that IAA and CK play the most direct and opposing roles: IAA inhibits axillary bud growth [34], whereas CK promotes bud outgrowth [35]. IAA is synthesized in the shoot apex and transported basipetally, thereby maintaining apical dominance and suppressing the growth of lower axillary buds [36]. A reduction in IAA levels or disruption of its transport weakens apical dominance and triggers bud activation [37]. CKs are mainly synthesized in the roots and transported upward via the xylem; they antagonize IAA to promote the transition of axillary buds from dormancy to activation [38]. Moreover, CKs induce the expression of the WUSCHEL-related gene *OsTAB1*, thereby promoting the formation of axillary meristems and enhancing tillering potential [39]. In contrast, ABA accumulates in rice tiller buds and is closely associated with the maintenance of bud dormancy. High expression of the ABA biosynthetic gene *OsNCED1* enhances dormancy, while interactions between the ABA receptor *OsPYL* and the signaling component *OsPP2C* contribute to the suppression of lateral bud growth [40].

**Figure 8:**
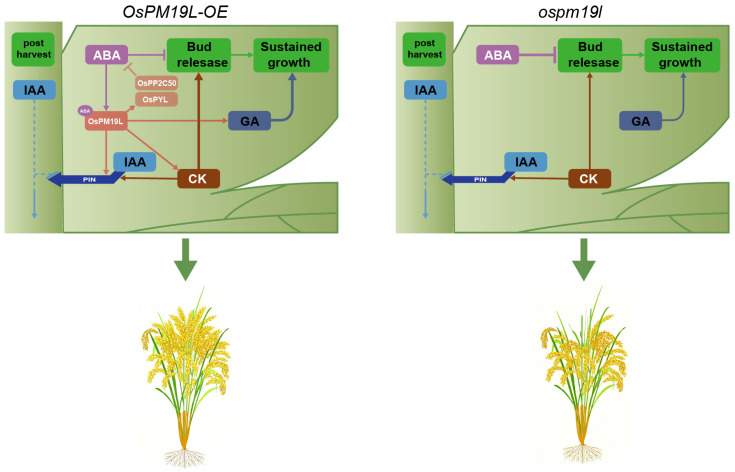
Hypothetical model of *OsPM19L* function in hormone regulation and axillary bud outgrowth after harvest in rice. *OsPM19L* is proposed to participate in post-harvest hormonal regulation and may modulate ABA metabolism through a negative feedback mechanism, thereby altering the relative balance of multiple phytohormones and promoting axillary bud activation during the ratoon season. Arrowheads represent positive regulation; blunt arrows represent negative regulation; and dashed lines represent indirect regulation.

In this study, hormone profiling showed that before harvest, axillary buds of *OsPM19L-OE* plants contained elevated levels of CKs and GAs, creating a hormonal environment favorable for bud activation. Compared with *ospm19l*, CK and GA levels in *OsPM19L-OE* axillary buds decreased markedly after harvest, which may indicate that these hormones were rapidly mobilized to support the release and early growth of dormant buds. Regarding IAA and ABA—both known to exert inhibitory effects on dormant bud outgrowth when present at high concentrations—their levels changed only modestly before and after harvest in the wild type. In contrast, both hormones increased significantly in *ospm19l* after harvest, whereas they decreased substantially in *OsPM19L-OE* (Figure 6). Expression patterns of key hormone-related genes further supported these findings: CK-degrading genes (*OsCKX9*, *OsCKX11*) and the ABA biosynthetic gene (*OsNCED1*) were upregulated in *ospm19l*, whereas the ABA receptor *OsPYL3* and the negative signaling regulator *OsPP2C50* were strongly induced in *OsPM19L-OE* (Figure 7). These results indicate that *OsPM19L* plays a role in the hormone-mediated regrowth of axillary buds during the ratoon season in rice. Based on these transcriptional profiles, we propose a putative feedback regulatory model: the *OsPM19L*-mediated influx of ABA may activate intracellular ABA signaling, thereby inducing the expression of key negative regulators in the ABA pathway (such as *OsPP2C50*) as well as ABA receptor genes (*OsPYL*). These transcriptional changes could, in turn, engage a negative feedback mechanism that modulates ABA biosynthesis and metabolism, ultimately resulting in a net decrease in ABA levels within axillary bud tissues (Figure 8).

It should be emphasized that drawing definitive conclusions regarding the role of *OsPM19L* in regulating a specific hormone is not warranted based solely on hormone measurements and gene expression data from a limited number of time points. Hormone levels are determined not only by gene expression but also by factors such as post-transcriptional regulation, translation efficiency, protein activity modification, and the potential involvement of other isoenzymes. Beyond the influence of *OsPM19L*, the hormonal fluctuations observed before and after harvest could also be associated with seasonal shifts in growth conditions, differences in carbohydrate mobilization, and stress-related physiological responses. Therefore, future studies should focus on identifying *OsPM19L*-interacting proteins and integrating transcriptomic, metabolomic, and protein–interaction analyses to more accurately delineate the regulatory network in which *OsPM19L* operates.

Collectively, *OsPM19L* orchestrates the dormancy-to-sprouting transition of axillary buds after harvest by modulating phytohormone levels, thereby enabling their rapid activation. Its dual roles—repressing tillering during the main season while promoting bud outgrowth in the ratoon season—reflect an adaptive mechanism for optimized resource allocation across growth phases. These findings provide new insights into the hormonal crosstalk underlying rice regeneration and offer potential molecular targets for breeding high-regeneration, high-yield ratoon rice varieties.

## Data Availability

The original contributions presented in this study are included in the article/Supplementary Material. Further inquiries can be directed to the corresponding authors.

## Supplementary Materials

The following supporting information can be downloaded at: https://www.mdpi.com/article/10.3390/plants14243843/s1, Figure S1. Construction and identification of transgenic materials. Figure S2. Phylogenetic tree and conserved motif analysis of PM19L proteins from various plant species. Figure S3. Transient GUS staining assay under ABA treatment. Supplementary Table S1. Primers used in this study. Supplementary Table S2. Genes investigated in this study.
