# Supplementary material for: OsPM19L Coordinates Phytohormone Signaling to Regulate Axillary Bud Outgrowth and Regeneration in Ratoon Rice
Source: Plants (Basel). 2025 Dec 17;14(24):3843. doi: 10.3390/plants14243843 (PMC12737186; doi:10.3390/plants14243843)
Supplement: Supplementary file 1 [file plants-14-03843-s001.zip › plants-3990220-supplementary.pdf]

Supplementary Figures

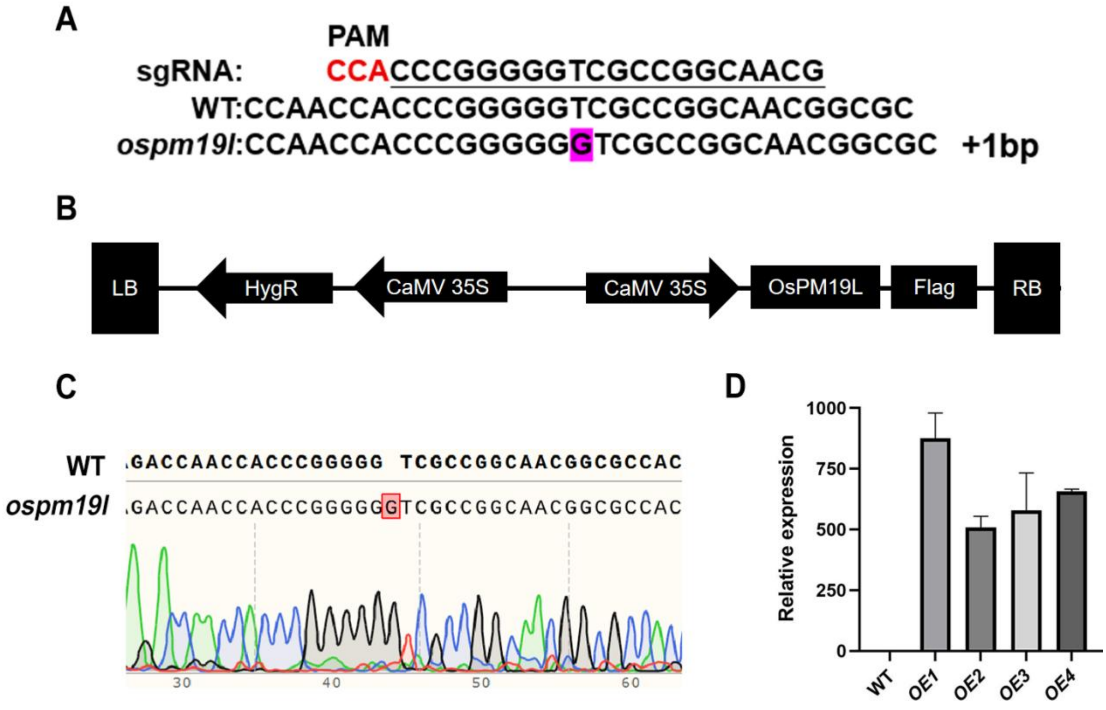

Figure S1. Construction and identification of transgenic materials.

(A) Target sequence of the *ospm19l* mutant.

(B) Schematic diagram of the vector used to generate the *OsPM19L-OE* lines. LB, left border; HygR, hygromycin phosphotransferase; CaMV 35S, cauliflower mosaic virus 35S promoter; Flag, epitope tag; RB, right border.

(C) Sequencing results of the *ospm19l* mutant, showing a frameshift mutation caused by the insertion of a single nucleotide.

(D) Relative expression level of *OsPM19L* in *OsPM19L-OE* lines. Data are means ± SD from three replicates.

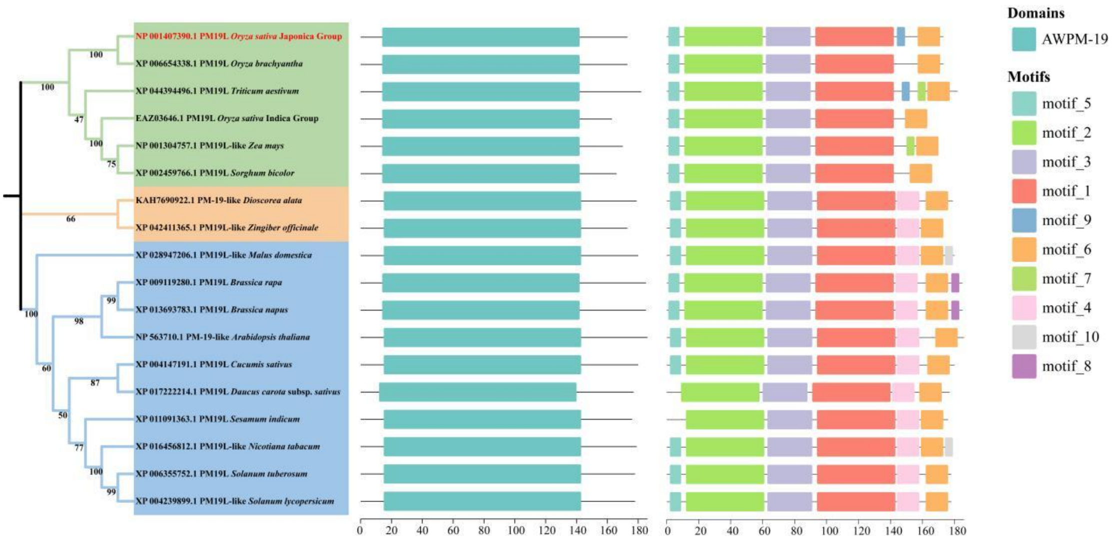

Figure S2. Phylogenetic tree and conserved motif analysis of PM19L proteins from various plant species.

The amino acid sequence of OsPM19L from Oryza sativa Japonica Group was used to identify homologous genes in other species through NCBI BLAST searches(BLAST: Basic Local Alignment Search Tool). The retrieved sequences were aligned using the ClustalW algorithm, and a phylogenetic tree was constructed with MEGA7 using the neighbor-joining (NJ) method with 1000 bootstrap replicates. As shown in the figure, species within the Poaceae family cluster together on a single branch, suggesting that PM19L genes in grasses may originate from a common ancestor and are evolutionarily conserved.

Conserved domain prediction using the NCBI revealed that all analyzed PM19L proteins contain the AWPM-19 domain, indicating strong evolutionary conservation and suggesting that these genes may share similar biological functions.

Motif analysis was performed using the MEME Suite(Introduction - MEME Suite), and the results showed that all species possess motif 1, motif 2, and motif 3. Based on the amino acid positions, these three motifs likely represent conserved substructures within the AWPM-19 domain. In addition, PM19L proteins from Poaceae lack motif 4, and motifs 7–10 exhibit species-specific variation, implying potential functional divergence of PM19L genes among different taxa.

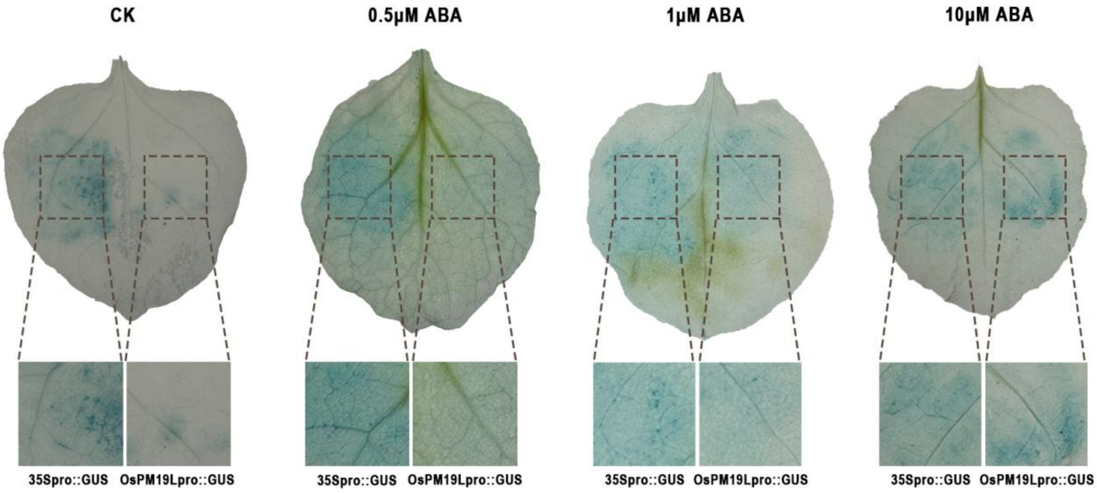

Figure S3. Transient GUS staining assay under ABA treatment.

Agroinfiltration was performed in *Nicotiana benthamiana* leaves, with 35Spro::GUS introduced into the left half of each leaf as an empty control and OsPM19Lpro::GUS introduced into the right half as the experimental treatment. Transformed leaves were then treated with four different concentrations of ABA for 24 hours, followed by GUS staining. Three biological replicates were included for each treatment. The staining intensity of the experimental group increased progressively with higher ABA concentrations, indicating ABA-responsive activation of the OsPM19L promoter.

Supplementary Table S1 Primers used in this study.

Primer (Forward)	Primer sequence	Primer (Reverse)	Primer sequence
**Construction**			
OsPM19-OE-F	AGAGAACACGGGGGACTCTTGCCAC CATGGCCGGAGTAGGGA	OsPM19-OE-	TCATCCTTGTAATCGCTGCCGCCGGA GACCCTGGCCGCGGCG
SgRNA-ospm19l-F	GATGGCCCAGGCGATGAG	SgRNA-ospm19l	TTGATCGTGATCGGGTTCGC
PR101-OsPM19L-EGFP-F	TTGATACATATGCCCGTCGACATGGC CGGAGTAGGGAGGA	PR101-OsPM19L-EGFP-R	GCTCACCATGGATCCGGTACCGACC CTGGCCGCGGCGGCG
3301-OsPM19L-PRO-F	GACCTGCAGGCATGCAAGCTTGGAG GACGTGACCGATTCA	3301-OsPM19L-PRO-F	TTACCCTCAGATCTACCATGGCGTGC TGGTTGGTGGAG
**RT-qPCR**			
Actin-F	AGCAGCATGAAGATCAAGGTGGTC	Actin-R	CCTTGGCAATCCACATCTGCTG
OsPM19L-qP-F	ACTACATCAACGGCGAGACC	OsPM19L-qP-R	CGAAGACGAGGAAGTAGAAGGTG
OsNCED1-qp-F	AAAGGATTTGCCCTGCTCAC	OsNCED1-qp-R	AGGGATGTAGGAAAGCCTCATG
OsPYL-qp-F	CAGGGACGCTTGTGATTGAATC	OsPYL-qp-R	GGCGAGAGATGTTAAGTTGCAC
OsPP2C50-qp-F	AAGAACAATGGCGCTGCATC	OsPP2C50-qp-R	TATCCTCGCTGCCTTTCTTCAG
OsYUCCA4-qp-F	AAGGGAAATGCGTGGAAAGG	OsYUCCA4-qp-R	TCCTGCACAATGTTGTTGGC
OsIAA3-qp-F	TAGTACGTGCAAGAGGTTGAGG	OsIAA3-qp-R	TTAACCCCTTGTTGCTCCTAGG
OsCKX11-qp-F	TCATCTACCCCATGCTCAAGTC	OsCKX11-qp-R	AGGTAGAAGATCTCGCCATTCG
OsCKX9-qp-F	ATGGCCATTTCAGCTTCCAC	OsCKX9-qp-R	ATGGTTGTGGCAATGTCAGC
OsSLR1-qp-F	TTCTGCACGCCCACTTCTAC	OsSLR1-qp-R	ATGGCTTGATTTGCGGTGAAG

Supplementary Table S2 Genes investigated in this study.

Gene Symbol	RAP-DB Locus ID	Description
OsPM19L	Os05g0381400	*plasma membrane protein1*
OsNCED1	Os02g0704000	*9-cis-epoxycarotenoid dioxygenase*
OsPYL	Os02g0255500	*pyrabactin resistance-like abscisic acid receptor*
OsPP2C50	Os05g0537400	*clade A type 2C protein phosphatase*
OsYUCCA4	Os01g0224700	*YUCCA-Like gene 4*
OsIAA3	Os01g0231000	*Auxin-responsive Aux/IAA gene family member*
OsCKX11	Os01g0231000	*cytokinin oxidase/dehydrogenase*
OsCKX9	Os05g0374200	*cytokinin oxidase/dehydrogenase*
OsSLR1	Os03g0707600	*slender rice 1*
